# On the Effect of Microwave Heating on Quality Characteristics and Functional Properties of Persimmon Juice and Its Residue

**DOI:** 10.3390/foods10112650

**Published:** 2021-11-01

**Authors:** Sofia Lalou, Stella A. Ordoudi, Fani Th. Mantzouridou

**Affiliations:** 1 Laboratory of Food Chemistry and Technology, School of Chemistry, Aristotle University of Thessaloniki, 54124 Thessaloniki, Greece; lalousofia@gmail.com; 2 Natural Products Research Center of Excellence (NatPro-AUTH), Center for Interdisciplinary Research and Innovation (CIRI-AUTH), 57001 Thessaloniki, Greece

**Keywords:** persimmon, *Diospyros kaki* Jiro, juice, solid residue, microwave heating, carotenoids, DPPH^●^ scavenging activity, polyphenols, pectin, color

## Abstract

In this study, it was investigated whether integration of microwave-heating into the pretreatment step of persimmon juice processing allows the concomitant production of both functional juice and added-value solid residue from the *Diospyros Kaki* “Jiro” cultivar. In this direction, persimmon pulp was treated under three different microwave-heating conditions (0.7, 4.2, and 8.4 kJ/g) prior to enzymatic maceration and compared to the non-heated material. Irrespective of microwave energy employed, the proposed hybrid treatment was highly efficient in terms of juice yield (70% *w*/*w*). The mildest heating conditions resulted in juice and residue that were both of inferior quality. Intensification of the microwave energy reduced the microbial load of the juice up to 2-log without compromising the content in total soluble solids, sugars, and L-ascorbic acid. Under the most drastic conditions, the juice was enriched in gallic acid, polyphenols, and potent DPPH^●^ scavengers, but its orange color faded and was more acidic. In parallel, the solid juice residue retained pro-vitamin A carotenoids (~278 µg retinol activity equivalents) and low-methoxy pectin (9 g/100 g DW). Overall, our findings can assist the efforts of the local juice processing industry to utilize persimmon fruits through energy-efficient technologies in a sustainable approach.

## 1. Introduction

The edible fruits of *Diospyros kaki* L. tree species, widely known as persimmon, have been highly appreciated in East Asian countries for their medicinal value since ancient times. Traditional Chinese and Japanese remedies refer to the consumption of fresh fruits or processed products (dried, juice, and vinegar) for alleviating a number of immune system and cardiovascular disorders [1]. Over the last decades, it was shown that the ripe fruits (peel and flesh) are rich in carotenoids with pro-vitamin A activity (e.g., β-cryptoxanthin, β-carotene), vitamin C, antioxidant polyphenols, tannins, and fibers [2], which altogether account for high nutritional value. The non-astringent “Hana Fuyu”, “Mopan”, and “Jiro” cultivars are among the most popular in China, South Korea, and Japan, the major producers and domestic consumers of persimmon in the world [2]. In Europe, Spain holds the greatest share in global exports with the astringent cultivar “Rojo Brillante” [3]. In the last decade, cultivation of the non-astringent “Jiro” and “Hana Fuyu” persimmon has been gaining importance in Northern Greece, especially in the Region of Central Macedonia, where major fruit tree plantations and industrial processing units of the country exist [4,5].

Currently, the product is destined for the domestic fresh fruit market, where it usually suffers great losses owing to non-homogeneous post-harvest ripening, but also because its seasonality coincides with that of other popular fruits. Thus, industrial fruit processing is in demand [6,7] to valorize surpluses and commercialize new added value products in order to sustain the fruit production system. Among the potential products, fresh juice attracts the interest of the functional juice market, but its production is very limited [7,8]. Persimmon juice production is suggested as a sensible as well as technically and economically valid utilization strategy that, however, does not eliminate the perishable nature of the fruits. Moreover, nutrients may be partially lost and a considerable amount of waste (skin, peduncles, and pulp residue) is generated during conventional juicing processes [9,10]. So far, strategies to minimize the discarded amounts of persimmon peel and pulp during industrial production of juice have not been extensively studied. Recently, it was shown that these streams are good sources of natural carotenoids with pro-vitamin A activity [9]. Carotenoids are widely used coloring agents and health-promoting, valuable ingredients for different end-use industries like food/beverage, animal feed, personal care/cosmetics, and pharmaceuticals [11]. Currently, they constitute a market segment with an increasing growth rate that is estimated to reach 1083.4 million USD by the end of 2026 [12]. The potential for recovering carotenoids and other valuable constituents such as pectin from persimmon juice residues deserves further attention for exploitation as it could be in line with the Sustainable Development Goal 12 of the 2030 Agenda [9,13,14].

Nowadays, the non-conventional green heating treatment that employs microwaves (MW) seems advantageous owing to its industrial adaptability, easiness of operation, minimum cost, low energy consumption, and high retention of bioactive compounds [10,15,16]. MW energy induces dielectric heating caused by the rotation of dipoles (such as water) in foods and brings about disruption of the cell walls [15]. Considering its application in key operations during the juice processing of several fruits, it is suggested to increase the juice yield and to aid in microbial and enzyme inactivation [16]. Some researchers have reported that using the microwave energy for fruit treatment (e.g., in mash or pulp) improved the quality characteristics of the resulting apple, plum, and blackberry juices [17,18,19]. MW-heating of juice residues like the peels of passion fruits has been also suggested to facilitate the recovery of valuable ingredients [20]. However, it is still not understood how integration of MW-heating in the pretreatment step of persimmon juice processing may affect the quality characteristics and functional ingredients of both the juice product and its residue.

**Figure 1:**
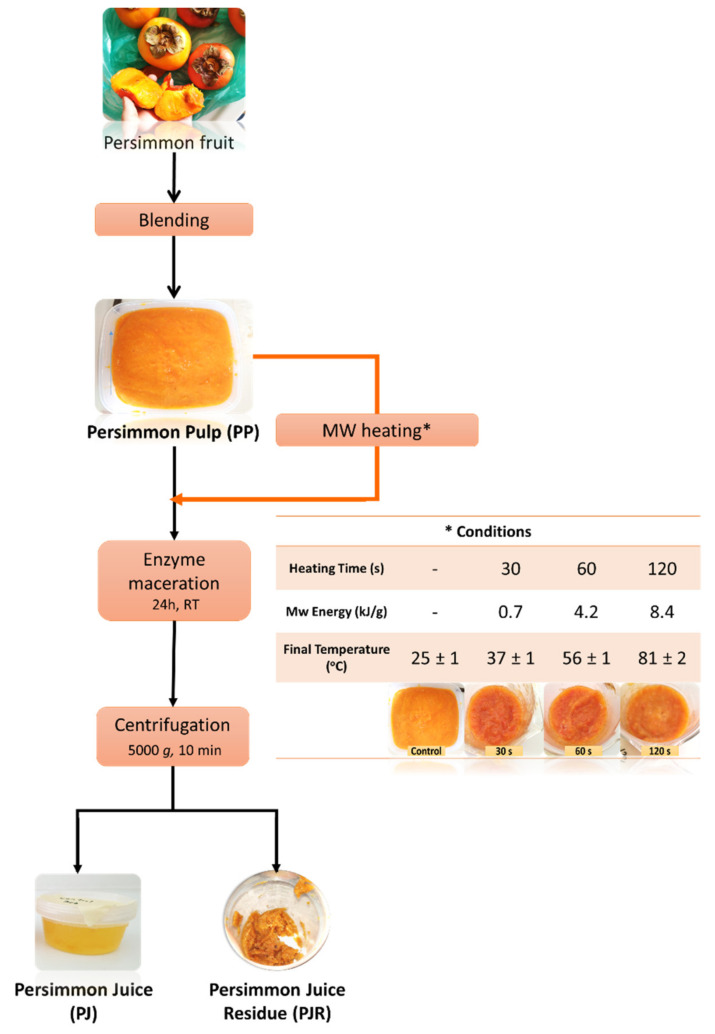
Flow diagram of persimmon juice processing without (black line) or after persimmon pulp treatment at different microwave (MW) heating conditions (red line) (the inset table shows the tested MW-heating conditions) (* the inset table shows the tested MW-heating conditions).

The present study investigates this prospect for the concomitant production of functional juice and added-value solid residue from persimmon fruits of “Jiro” cultivar (Figure 1). The juice yield, its microbial load, color, contents in soluble sugars, organic acids, vitamin C, and phenolic compounds along with the reducing activity toward the Folin-Ciocalteau reagent and DPPH^●^ scavenging activity were evaluated prior to and after employing different MW-heating conditions (MW power and time/temperature conditions). In parallel, the solid juice residue was assessed as a potential source of pro-vitamin A carotenoids and pectin. To the best of our knowledge, such an integrated approach for processing persimmon juice and its residue is presented for the first time in the literature.

## 2. Materials and Methods

### 2.1. Chemicals and Reagents

All chemicals and reagents used in this study were of analytical or HPLC grade. Folin–Ciocalteu’s phenol reagent, sodium carbonate (Na_2_CO_3_), sodium chloride (NaCl), ascorbic acid, 1,1-diphenyl-2- picrylhydrazyl (DPPH^●^), and potassium hydroxide (KOH) were purchased from Sigma-Aldrich (Steinheim, Germany). Gallic acid was provided by Merck (Darmstadt, Germany). β-carotene (β-Car) (≥95% purity by HPLC, DRE-CA11045800) and lycopene analytical standard were from LGC standards (Wesel, Germany), while β-cryptoxanthin (β-Cry) (97%, FC57753) and zeaxanthin (≥95%) were from Carbosynth (Bratislava, Slovakia). Lutein (≥88%) was from Glentham (Corsham, UK). High retention seamless cellulose dialysis tubing (MWCO 12400) was purchased from Sigma-Aldrich (Steinheim, Germany). Plate Count Agar (PCA), Potato Dextrose agar (PDA), and Violet Red Bile Glucose Agar (VRBGA) were all acquired by Lab M Limited (Heywood, UK). Selenite cystine broth base, USP, and Salmonella Shigella (SS) Agar (modified) were purchased from Biolab (Budapest, Hungary). Butylated hydroxytoluene (BHT) (≥99%,), glucose, fructose, ascorbic acid, acetic acid, citric acid, and malic acid were all purchased from Sigma-Aldrich (Steinheim, Germany), while ammonium carbonate and sodium sulphate were provided by PanreacQuimica (Barcelona, Spain). Solvents used in HPLC analyses—namely, hexane, methyl-tert-butyl ether (MTBE), acetone, ethanol, methanol, and water—were all purchased from Sigma Co. (St. Louis, MO, USA). Low-methoxy commercial citrus pectin was provided by Pomona’s Universal Pectin (Oakhurst, CA, USA). FT-IR grade KBr (>99%) was purchased from Sigma-Aldrich (Steinheim, Germany).

### 2.2. Material and Sample Preparation

Persimmon fruits of the commercial cultivar “Jiro” from the region of Imathia (Naousa, Greece), and of good physical integrity, were purchased from a local market in Thessaloniki during late November 2020. The fruits (approximately 20 kg) were allowed to ripen in the dark and at ambient temperature until the ready-to-eat stage (orange-reddish in color). The ripened fruits were washed thoroughly with tap water, drained, left to air dry, and then cut in halves. Each piece was cleaned from the woody peduncles, cut in halves again, and subsequently ground into thick slurry with the aid of a household blender (ROHNSON R-516, PRC). The persimmon pulp (PP) sample, immediately after its preparation, was divided into parts of 500 or 900 g each and stored at −18 °C in BPA-free plastic containers with airtight lids until further processing (maximum 48 h). The PP was dried at 105 °C until constant weight to determine the percent moisture content (% *w*/*w*).

### 2.3. Microwave Heating

MW-heating tests were carried out using a household microwave oven (TCL, 700 Watt). Briefly, a 50 g fraction of homogenized PP that was pre-conditioned at 25 ± 2 °C was placed in a pyrex beaker (H 9 cm, Ø 7 cm) and exposed to a preselected MW power values (210 W) for different times (30, 60, and 120 s) (Figure 1). The above heating conditions were selected based on preliminary tests (Supplementary Materials, Table S1) that involved calculation of the MW energy delivered to the sample and direct measurement of the PP core temperature achieved at the end of the heating treatment. MW energy values were calculated using the equation E = Wt/m, where W is microwave oven power, t is time of MW exposure, and m equals the quantity of sample [19]. Under the employed conditions of MW energy (0.7, 4.2, and 8.4 KJ/g), the final core temperature ranged from 30 to 80 °C, allowing to evaluate various attributes (microbial load, color, antioxidants, carotenoids, and pectin) that are affected differently by temperature and to avoid PP overheating or burning.

### 2.4. Persimmon Juice Preparation

The MW-heated pulp was subsequently macerated for 24 h with 112 mg/kg macerating enzyme pectinase (Viazym Clarif Plus P, Martin Vialatte, France), at room temperature. Immediately after maceration, the MW-heated pulp was placed in a water bath at 95 ± 2 °C for 10 min to ensure full inactivation of the macerating enzyme. The mixture was removed from the water bath and immediately dipped into iced water. Non-MW-heated pulp was also treated enzymatically and considered as control in this study (PJ-C). PP without any treatment was also tested.

Then, the mixture was centrifuged at 5000× *g* for 10 min in order to separate persimmon juice (PJ) from the persimmon juice residue (PJR) (Figure 1). Both samples were sealed in plastic containers and stored at −18 °C until further treatment and/or analysis. Each juicing process was performed in triplicate. The juice yield was calculated as g juice per 100 g PP.

### 2.5. Tests on Persimmon Juice

#### 2.5.1. Microbiological Analysis

Samples of PP (1 g) and PJ (1 mL) were serially diluted (10-fold) in sterilized Ringer solution and plated by the spread plate technique on appropriate media for microbiological examination. Aerobic mesophilic counts (AMCs) were enumerated on Plate Count Agar (PCA) after incubation at 30 ± 1 °C for 48 h; yeasts and molds counts (YMCs) were enumerated on Potato Dextrose Agar (PDA) after incubation at 25 °C for 2–7 d. For the enumeration of Enterobacteriaceae and coliforms, VRBGA and VRBA were used, respectively (overlay; incubation at 37 ◦C for 24 h). For the determination of Salmonella, 25 g of persimmon were transferred to a stomacher bag with 225 mL of selenite cystine broth base and incubated at 37 °C for 18–24 h. Then, the enrichment mixture was plated on SS Agar (incubation at 37 °C for 24 h) [21]. The results from plate counts were expressed as log_10_ colony forming units (CFUs)/g or mL.

#### 2.5.2. Total Soluble Solids and Individual Sugars

Total soluble solids (TSSs) were determined using a handheld refractometer (Schmidt & Haensch, GmbH & Co., Berlin, Germany) and expressed as °Brix. Glucose and fructose were quantified using HPLC-RID, as described by Lalou et al. [22]. Briefly, the aforementioned sugars were separated on a hydrogen form cation exchange resin-based column Agilent HI-plex H (Agilent Technologies, Santa Clara, CA, USA) by isocratic elution (5 mM sulfuric acid solution at a flow rate of 0.5 mL/min). The samples were properly diluted in the mobile phase and filtered through 0.45 mm pore size regenerated cellulose membrane filters (Schleicher & Schuell, Dassel, Germany). The column was equilibrated in an oven at 65 °C and the injection volume was 10 mL. The HPLC system was composed of an LC-10Advp pump (Shimadzu, Kyoto, Japan) and a refractive index detector (RID-6A, Shimadzu, Kyoto, Japan). The Clarity Chromatography Software v8.2 (DataApex, Prague, Czech Republic) was used for data processing. Quantification was made using external calibration curves for standard glucose and fructose in the range of 2.5–60 g/L.

#### 2.5.3. Total Acidity, pH, and Organic Acids’ Composition 

Total acidity (TA) was determined according to AOAC official titrimetric method (AOAC method 982.27) [23] and pH was measured with a MP220 pH Meter (Mettler-Toledo, Greifensee, Zurich, Switzerland). Chromatographic analysis of individual organic acids was carried out using an HPLC chromatographic system composed of an LC-20AD pump and a UV/Vis SPD-10AV detector (Shimadzu, Kyoto, Japan). Separation was achieved on a cation exchange resin-based column Agilent Hi-plex H with an aqueous 10 mM sulphuric acid solution and a flow rate of 0.6 mL/min. The oven temperature was 50 °C and the injected volume was 10 µL. The Clarity Chromatography Software v8.2 (DataApex, Prague, Czech Republic) was used for data processing. The monitoring wavelength was set at 210 nm. Peak identification was carried out by comparison of their retention times with those of standard compounds. External standard calibration curves were prepared using solutions of citric, tartaric, malic, and acetic acids in the range of 0.5–5.0 g/L for quantification of each compound, and the results were expressed as g/L of PJ. 

#### 2.5.4. Color Evaluation

Color was objectively measured with the aid of a portable HunterLab spectrophotometer (MiniScanTM XE Plus, Reston, VA, USA) and with reference to the D65 illuminant source and the 10° Observer. For this purpose, an aliquot of each juice sample was placed in a glass sample cap (Ø 64 mm) and reflectance measurements were taken against a white and a black background. The data were automatically transformed according to the CIELAB color system, so that tristimulus coordinate L, a*, and b* values, expressing lightness (L*), variation between red/green (positive vs. negative a* values), or yellow/blue color (positive vs. negative b* values), could be to be recorded. Chroma (C*) and hue (h*) values were also calculated [24]. For intra-day repeatability assessment, eleven replicates per sample were examined (CV = 7.5, *n* = 11). The color differences ($\Delta E_{ab}^{*}$) between the PJ-C and PJ samples after MW-heating of the PP were calculated as the Euclidean distance between two points in the three-dimensional space defined by L*, a*, and b* using the following formula [25]:

$$\Delta E_{\mathrm{ab}}^{*}=\sqrt{{\left(\Delta L\right)}^{2}+{\left(\Delta a^{*}\right)}^{2}+{\left(\Delta b^{*}\right)}^{2}}$$

where ΔL*, Δa*, and Δb* are differences between the control persimmon juice (PJ-C) and PJ samples after MW-heating of the PP. 

#### 2.5.5. L-Ascorbic Acid Determination

Ascorbic acid content of PJ samples was determined according to AOAC official titrimetric method (AOAC method 967.21) [26]. All determinations were performed in triplicate at room temperature, and the results are expressed as mg ascorbic acid/L of PJ.

#### 2.5.6. Preparation of Phenolic-Rich Extracts 

Polar phenolic compounds were extracted from PJ samples according to Ubeda et al. [27]. Briefly, a portion of about 5 g (± 1 mg) of PJ was successively extracted three times with 20 mL of methanol/water (80:20), sonicated for 15 min in an ultrasonic bath (Ultrasonic Cleaner-VWR, Bridgeport,, PA, USA), and centrifuged (1800 *g*, 10 min, 4 °C). The cleaned extracts were combined and the solvent was evaporated under vacuum (Buchi, Rotavapor, Model 100, 45 °C). The dry residue was re-dissolved in 5 ml of methanol/water (80:20), filtered through a cellulose syringe filter (0.45 µm), and kept at −20 °C for a maximum of 12 h prior to analysis. Three replicate extracts per PJ sample were prepared.

#### 2.5.7. Folin–Ciocalteu (F–C) Assay

Owing to the expected complexity of juice composition, the F–C assay was used to measure antioxidant activity [28]. The F–C assay was applied to juice extracts as in a previous study [24]. Briefly, suitable aliquots of the polar extracts were transferred into a 10 mL volumetric flask and water (5 mL) and the Folin–Ciocalteu reagent (0.5 mL) were added. After 3 min, 1 mL of saturated (37%, *w*/*v*) sodium carbonate solution was added to the reaction mixture. The solution was diluted with water to 10 mL and the absorbance at 725 nm was measured after 1 h against a blank solution with a spectrophotometer UV-1601 (Shimadzu Co., Kyoto, Japan). Gallic acid was used as an external standard and the results were expressed as gallic acid equivalents (mg GAE/L PJ). All determinations were performed in triplicate at room temperature.

#### 2.5.8. RP-UHPLC-DAD-FLD Analysis of Phenolic Compounds

The RP-UHPLC profile of phenolic compounds was examined on a Shimadzu Nexera X2 UHPLC System (Shimadzu Corporation, Kyoto, Japan), equipped with a LC-30AD pump, a SIL-30AC autosampler (50 μL loop), a CTO-20AC column oven, and a UV/visible diode array SPD-M30A detector (DAD) coupled to a RF-20AXS fluorescence detector (FLD). Data acquisition and analysis were carried out using Lab Solution ver. 5.86 software (Shimadzu Corporation, Kyoto, Japan). Separation was achieved on a 75 mm × 2.0 mm, 1.6 m, Shim-pack XR-ODS III Shimadzu chromatographic column (Shimadzu, Kyoto, Japan), according to the method of Tsimidou et al. [29]. The mobile phase consisted of water (0.2% phosphoric acid) (A), methanol (B), and acetonitrile (C) at a flow rate 0.45 mL/min. The gradient elution program was as follows: 0–11 min, 2–25% B and 2–25% C; 11–13 min, 25–30% B and 25–30% C; 13–17 min, 30–50% B and 30–50% C; 17–20 min, 50% B and 50% C; 20–20.5 min, 50–2% B and 50–2% C; and 20.5–28 min 2% B and 2% C. Equilibration was from 28 to 30 min. Column temperature was maintained at 40 °C and that of the sample at 6 °C. Injection volume was either 5 µL for extracts or 10 µL for standard solutions (gallic acid). Chromatograms were recorded at 280 nm (UV) and at 280 nm (exc.)/320 nm (em.) (hydroxycinnamic acid derivatives), 280 nm (exc.)/339 nm (em.) (catechin analogues) (FL) [24,25]. Peak identification was based on available standards, relative retention times, spectra matching, and the literature [30,31]. The external standard calibration curve was prepared using stock methanolic solution of gallic acid in the range of 2–50 mg/Kg.

#### 2.5.9. DPPH^●^ Scavenging Assay

Radical scavenging activity (RSA) of PJ samples was determined photometrically using the DPPH^●^ assay, as previously described [24]. Each sample was analyzed directly, without prior dilution or extraction, in triplicate. The results were the mean value of three measurements and were expressed as % RSA = [(A516 (t = 0 min) − A516 (t = 30 min)) × 100/A516 (t = 0 min)]. All determinations were performed in triplicate at room temperature.

### 2.6. Tests on Persimmon Juice Residue

#### 2.6.1. Extraction and Purification of Pectin 

The extraction and purification of pectin were performed according to Jiang et al. [14] with minor modifications. A small amount (~2.5 g) of oven-dried PP or PJR (60 °C, 24 h) was mixed with a 20-fold volume of deionized water. For the hot acid extraction, the pH of the mixture was adjusted to 2.0 using 1 M citric acid solution and was placed in a 90 °C water bath for 120 min with constant stirring at 500 rpm. The supernatant was diluted with twofold anhydrous ethanol, stirred gently for 15 min, and placed at 4 °C overnight for precipitation. Crude pectin was obtained by centrifugation at 4500× *g* for 10 min, washed with 95% (*v*/*v*) ethanol, and then dialyzed with a dialysis tube (MW 12,400 cut off) for 24 h to remove impurities. Finally, the purified pectin was obtained by freeze-drying. The pectin yield was expressed as g per 100 g PP or PJR FW or DW.

Pectin purity was examined by transmittance FT-MIR spectroscopy using a Shimadzu IRAffnity-1 (Shimadzu Europa GmbH, Duisburg, Germany) spectrometer. The pectin sample (5 mg) was mixed with KBr (180 mg) for disc preparation (in triplicate). Two spectra per each disc were acquired in the absorbance mode, each with 100 scans at a resolution of 4 cm^‒1^ and within the mid-infrared frequency range of 4000–400 cm^‒1^. Spectra were stored and pre-processed using the software IRsolution (v. 1.50) supplied by the same manufacturer. The average absorption spectra were calculated after smoothing by Savintzky‒Golay method (15 data points) and manual baseline correction between 1824.7 and 1573.9 cm^−1^. The methyl-esterification degree (MED) of pectin was calculated as the mean ratio of the peak area at around 1730 cm^−1^ (carboxylic ester bonds) over the sum of the peak areas of 1730 cm^−1^ and around 1630 cm^−1^ (carboxylate ion moieties) [32]. 

#### 2.6.2. Preparation of Carotenoid-Rich Extracts 

The carotenoid-rich extracts were prepared according to the protocol described by Bordiga et al. [33], with slight modifications. In particular, a portion of about 5 g (±1 mg) of wet sample (PP or PJR) was accurately weighed in a conical flask and mixed with 30 mL of acetone containing BHT (0.1% *w*/*v*) as antioxidant and ammonium carbonate (0.1% *w*/*v*) as neutralizer. The sample was sonicated in an ice water bath sonicator for 5 min and then centrifuged (4500× *g*, 15 min). The same procedure was repeated until a total volume of 100 mL of acetone fraction was collected. This fraction was subsequently transferred in a separation funnel with 100 mL of a mixture of hexane/diethyl ether (1: 1 *v*/*v*) and 100 mL of a 10% *w*/*v* aqueous sodium chloride solution. The organic layer was first dried over anhydrous sodium sulphate for 10 min and then evaporated to dryness in a rotary evaporator (35 °C). The extraction procedure was performed in duplicate. The intra-day variability of extraction per each sample batch (CV%, *n* = 3) was found to be below 11.5%. Each replicate was subsequently analyzed by HPLC-DAD. Prior to injection, the carotenoid‒rich fraction was dissolved in 2 mL of a mixture of MeOH/MTBE/H_2_O (45.5: 52.5: 2 *v*/*v*/*v*) and filtered through a 0.45 μm pore membrane filter.

#### 2.6.3. Total Carotenoid Content Estimation

The dry extract was re-dissolved in acetone and its content in total carotenoids was assessed after reading the absorbance values at 450 nm. A calibration curve was obtained using β-Cry as the standard. The results were expressed as mg of β-Cry/100 g PJR (FW).

#### 2.6.4. Carotenoid Profiling by HPLC‒DAD

Analyses of carotenoids were performed by HPLC using an ECOM spol. s r.o., Czech Republic system (model ECS05). The system is comprised of a quaternary gradient pump (ECP2010H), a gradient box with degasser (ECB2004), a column heating/cooling oven (ECO 2080), an autosampler (ECOM Alias), and a diode array detector (ECDA2800 UV‒VIS PDA Detector). Carotenoids were separated on a C30 reverse phase column (YMC‒Pack YMC C30, 250 mm × 4.6 mm id, S‒5 μm, YMC Co., Ltd), under elution conditions described previously [34,35]. Briefly, a linear gradient change in eluent composition from 100% A (MeOH/MTBE/H_2_O, 81: 14: 4, *v*/*v*/*v*) to 100% B (MeOH/MTBE, 10:90, *v*/*v*) was achieved within 60 min, and then back to the initial conditions in 10 min. The flow rate was set at 1 mL/min, the column temperature at 32 °C, and the injection chamber at 4 °C. The injection volume was 5 μL for extracts and 10 μL for standard compounds.

Monitoring wavelengths were set at 450 and 402 nm (for *cis*-carotenoids), as previously suggested [36]. The UV/Vis spectra between 220 and 700 nm were also acquired and the chromatograms were processed at 450 nm with the aid of the Clarity Chromatography Software v8.2 (DataApex Ltd. Prague, Czech Republic). Individual carotenoids present in PP and PJR were tentatively identified on the basis of UV/Vis spectral characteristics (λmax), retention time, and elution order on C30 column. These data were evaluated also considering the performance of available reference compounds as well as the respective HPLC‒DAD profile reported recently by Cano and co-workers [34,35] for carotenoids of freeze-dried persimmon pulp under identical elution conditions. The identity of the major esterified carotenoids was also investigated by LC-QTOF-MS/MS (APCI^+^) analyses under conditions described in the Supplementary Material. 

External standard calibration curves were prepared using stock solutions of β-Cry and β-Car after appropriate dilutions with acetone (containing BHT 0.05%) or methanol, respectively. Linear five-point curves were constructed with the least squares method in the concentration range of 2–25 µg/mL for β-Cry and 2–10 µg/mL for β-Car (R^2^ > 0.979). Mass was corrected according to the chromatographic purity of each standard. Each of the concentrations tested was analyzed in triplicate. The limit of detection (LOD, calculated as 3.3 s/b, where s is the standard deviation of the intercept value and b is the slope of the calibration graph) and limit of quantification (LOQ, calculated as 10 s/b) [29] for b-Cry were ≥3.3 and 11.1 ng, respectively. The LOD and LOQ values for b-Car were ≥9.1 ng and 30.4 ng, respectively. Molecular mass differences between free and esterified forms of β-Cry were corrected by dividing the mean molecular mass of the major esters (β-Cry-laurate, 735.2 amu; myristate, 763.2 amu) by the molecular mass of the free β-Cry (552.9 amu). The results were expressed as μg/100 g PP or PJR (FW or DW). 

For multivariate statistical analysis, the chromatograms of PP and PJR extracts were first aligned manually relative to the major peak of PP that eluted at t_R_ ~ 33.2 min and corresponds to the lauric acid monoester of (all-*E*)-β-cryptoxanthin. Each chromatographic peak or group of peaks presenting the carotenoid-specific fine structure in the spectral region between λ=410−470 nm, eluting between 5 and 45 min of each run, was integrated and the areas were normalized against the total chromatographic area at 450 nm. The elution time intervals of those distinct peaks were specified by recording the range of t_R_ value among replicate runs (see Table A1). The % of total peak area values were extracted to an Excel sheet with 62 columns—variables for time intervals at 450 nm and 5 rows for the samples under study (PP, PJR-C, PJR-M30, PJR-M60, PJR-M120). The 62 sub-regions of each chromatogram are defined with consecutive numbers in Table A1 (Appendix A).

### 2.7. Statistical Analysis

Data analysis (regression analyses, descriptive statistics) was carried out using the Microsoft Excel 13 software (Microsoft, Co., USA). Statistical comparison of the mean values was performed by one-way ANOVA, followed by the multiple Duncan test (*p* < 0.05 confidence level) using SPSS 22.0 software (SPSS Inc., Chicago, IL, USA). SIMCA® 16 (Umetrics, Umeå, Sweden) was used for the multivariate analysis of chromatographic data.

## 3. Results and Discussion

### 3.1. Persimmon Juice 

#### 3.1.1. Juice Yield and Physicochemical Characteristics

Monitoring the juice yield is essential for decision-making in juice processing plants [7]. In our study, only a slightly increase was evidenced after enzymatic maceration of PP compared with the untreated PP (36.6 ± 1.5 vs. 35.8 ± 4.0% *w*/*w*). MW-heating integrated into the pretreatment step of juice processing prior to enzymatic maceration almost doubled the process yield (Table 1). The latter reached nearly 70% *w*/*w* of the initial PP weight, even at the mildest MW-heating conditions tested. Our results imply a short of synergism between the applied pretreatments that favors pulp tissue softening, and thus the juice extraction yield. When the former treatments run independently, MW-heating even at the highest energy level was as efficient as the enzymatic treatment (data not shown), thus strengthening our approach to adopt a hybrid treatment. 

The physicochemical characteristics of the PJs are shown in Table 1. MW-heating for 60 to 120 s did not alter the TSS content of PJs, except for that of PJ-M30. Fructose and glucose were the most abundant sugars of PJs, as expected [7,37]. Quantitative differences among the samples (66–80 g/L for glucose and 78–91 g/L for fructose) were not considered appreciable. These values were slightly higher than those reported for persimmon juice obtained from other varieties, maturity levels of the raw material, and production processes [7]. It has been reported that juices produced from MW-heated apple mash and plum pulp were significantly enriched in sugars with no qualitative changes [17,18]. In the present study, the glucose-to-fructose ratio, related to the intensity and the persistency of the juice sweetness [22] decreased significantly in PJs from MW-heated PP (0.80 to 0.88 vs. 0.96 for PJ-C).

According to the data in Table 1, PJs from MW-heated PP had an average pH value below 3.4, comparable to that of PJ-C and slightly higher TA values. Acetic, citric, tartaric, and malic acid were found to dominate the organic acid profile of all the studied PJs. The most intriguing finding here concerned the high relative concentration of acetic acid in PJs produced from MW-heated PP, especially at the most drastic condition (PJ-M120). The obtained values were much higher than those reported by other researchers for persimmon juice obtained with different processes, varieties, and ripening stages of persimmon (e.g., [7,38]). As pectin from persimmon peel is highly acetylated [14], it is possible that MW-heating of the PP under the low pH conditions favors the hydrolysis of pectin, with consequent deacetylation and liberation of free acetic acid moieties [39]. That being the case, and considering that the human detection threshold for acetic acid is quite low (∼0.1 mM ≡ 60 mg/L, ∼pH 4.4), it is expected that the PJs from MW-heated PP would be perceived as acidic. This is in line with the results reported for apricot and plum juices from MW-heated mash [40]. On the other hand, the contents of citric, tartaric, and malic acids were highest in PJ-M30, almost 1.5-fold higher than those in PJ-C. Intensification of MW-heating conditions negatively affected the content of these organic acids (Table 1). 

The color of the PJ samples was assessed in this study in terms of its tristimulus coordinates in the CIELa*b* color scale (Table 1). The PJ-C presented a bright vivid orange yellow color. It has been reported that differences in perceivable color can be analytically classified as not noticeable (0–0.5), slightly noticeable (0.5–1.5), noticeable (1.5–3.0), well visible (3.0–6.0), and great (6.0–12.0) [10]. The ΔE value of the PJ-M30 and PJ-M60 was equal to 4.08 and 4.90, respectively, signifying a well-visible difference in color that could be perceived by inexperienced observers [41]. In fact, the color of the juice became brighter (higher L values) and more yellowish (unaffected b*, higher h* values) compared with the PJ-C as the red hue disappeared (negative a* value). Extension of the MW-heating time up to 120 s brought about great visual change in the color of the corresponding PJ (ΔE_PJ-M120_ =11.1). So far, evaluation of the persimmon juice color in terms of CIEL*a*b* is scarcely discussed in the existing literature [6], thus the results can be hardly inter-comparable.

#### 3.1.2. Microbiological Quality and Safety

In the microflora of untreated PP, Enterobacteriaceae, coliforms, and Salmonella spp. were undetectable (<10 CFU/g). This finding indicates its safety and good hygienic status. Moreover, it showed moderate numbers of both AMCs (4.63 ± 0.02 log_10_CFU/g) and YMCs (3.91 ± 0.04 log_10_CFU/g).

Indicator microorganisms (Enterobacteriaceae, coliforms, and Salmonella) were not detected in any of the studied PJs. This complies with microbiological criteria for unpasteurized fruit and vegetable juices (ready-to-eat) [42].

**Figure 2:**
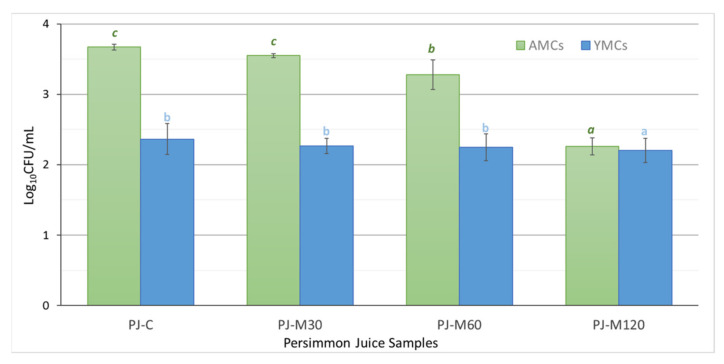
Changes in the population (log_10_CFU/mL) of aerobic mesophilic counts (AMCs) and yeasts and molds counts (YMCs) in persimmon juice produced without (PJ-C) and after persimmon pulp treatment at different microwave heating conditions (PJ-M30, PJ-M60, and PJ-M120). Error bars represent the standard deviation (sd) of the mean value (*n* = 4). Different lowercase letters in the same substrate indicate significant differences (*p* < 0.05).

Differences in the size of AMCs and YMCs content of PJs are presented in Figure 2. Regarding PJ-C, AMCs and YMCs values were lower than those of the untreated PP (3.6 ± 0.04 log_10_CFU/mL and 2.36 ± 0.22 log_10_CFU/mL, respectively), indicating that the microbial counts were affected to a degree by heating treatment conditions for the inactivation of the macerating enzyme. The molds counts were below the limit of detection (1.00 log_10_CFU/mL). Despite the fact that their acidic nature provides a natural safety barrier [43], a low initial microbial load guarantees prolonged shelf life and sensorial stability. Microbial levels exceeding 6 log_10_CFU/mL are considered extremely high and are associated with the use of low quality or deteriorated fruits, or poor sanitation and hygiene conditions during handling and processing [7].

By integrating MW-heating into the pretreatment step of persimmon juice processing, a clear trend was observed between the heating time and the microbial reduction achieved. The most pronounced effect was evidenced for AMCs in PJ-M120 with a 2-log reduction compared with the respective value in PJ-C. Reduction by 0.16 log (*p* < 0.05) was recorded for YMCs. Our finding concerning AMCs in PJ-M120 showed that heating under the longest time/highest MW energy conditions helped to achieve a similar content to that of juices pasteurized with non-conventional heating technologies [44,45]. Microorganisms are susceptible to MW energy owing to thermal and non-thermal effects. The latter is still a subject of ongoing research. In any case, the matrix should contain ionic molecules to enable microwave heating [16]. The most popular theory suggests the lethal effect of cell electroporation [15]. Thus, MW-heating in the pretreatment step of PJ processing can be considered as an effective means for microbial inactivation. In a cumulative microbial reduction process, starting from fruit pulp to final juice packaging, MW-heating is expected to reduce juice pasteurization requirements intended to control microbiological hazards. 

#### 3.1.3. L-Ascorbic Acid Content

**Figure 3:**
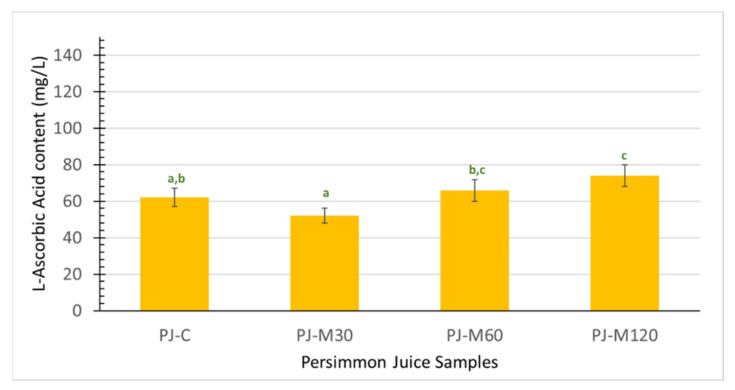
Changes in L-ascorbic acid content (mg/L) of persimmon juice produced without (PJ-C) and after persimmon pulp treatment at different microwave heating conditions (PJ-M30, PJ-M60, PJ-M120). Error bars represent the standard deviation (sd) of the mean value (*n* = 4). Different lowercase letters indicate significant differences (*p* < 0.05).

In our study, PJ-M120 presented the highest content of L-ascorbic acid (74 ± 6 mg/L) (Figure 3), comparable in size to those of the other PJs and indicating that MW-heating of PP did not have an appreciable effect on this parameter. Our observations are in line with those reported for MW-heated orange and grapefruit juices at intense conditions [10]. On the other hand, Zhang et al. [44] showed that vitamin C of apple juice from MW-heated mash was degraded over a range of different MW energy values. This finding suggests that the impact is likely dependent on the type of fruit, sample characteristics, employed conditions, and so on. Nonetheless, the obtained values in our study are higher than those reported for juices from MW-heated apple mash (13–48 mg/L) [44].

#### 3.1.4. Antioxidant Potential 

**Figure 4:**
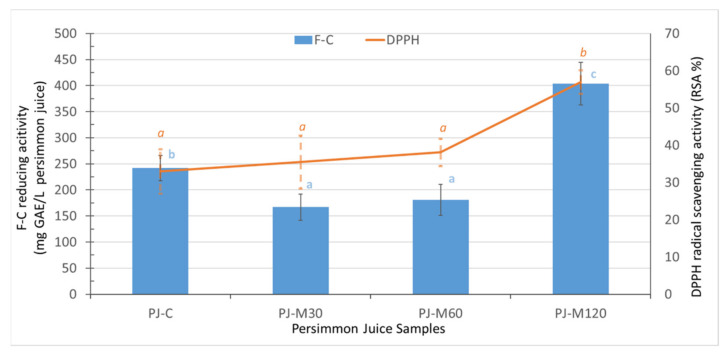
Antioxidant potential of persimmon juice without (PJ-C) and after persimmon pulp treatment at different microwave heating conditions (PJ-M30, PJ-M60, and PJ-M120 using the Folin–Ciocalteu (F–C) and DPPH^●^ assays. Error bars represent the standard deviation (sd) of the mean value (*n* = 4). Different lowercase letters in the same test indicate significant differences (*p* < 0.05).

The reactivity of phenolic-rich extracts of PJs toward the Folin–Ciocalteu reagent was tested to obtain a gross assessment of the reducing antioxidant potential of the hydrophilic constituents. The F–C values along with % RSA ones, representing radical scavenging activity antioxidant potential, are presented in Figure 4 and discussed as follows.

PJ-C exhibited 242 mg GAE/L, comparable to those reported for persimmon juices that underwent drastic heating treatments (304–350 mg/L) [7,46]. MW-heating of the PP at the shortest time-lowest MW energy condition (30 s/0.7 KJ/g) resulted in 30% lower F–C values in juice. The same effect was also evidenced after increasing the heating time to 60 s (Figure 3). Residual polyphenol oxidase activity in MW-heated PP of low final temperature may account for the evidenced decrease in F–C values of PJ-M30 and PJ-M60. More efficient thermal inactivation is expected to be achieved under more drastic MW-heating conditions [47]. This coincides with the fact that, when PP was MW-heated at the most intense conditions (120 s/8.4 KJ/g), the F–C value of PJ-M120 was 168% higher as compared with PJ-C. In addition, disruption of the cell walls was likely more extensive, allowing better release of redox-active phenolic compounds that are bound to the pectin, cellulose, hemicellulose, and lignin traces [15]. Exposure of PP to different MW-heating conditions also affected the radical scavenging activity of the resulting PJs. As shown in Figure 3, the %RSA values were slightly higher in PJ-M30 and PJ-M60 and >100% higher in PJ-M120, indicating that the release of potent, hydrophilic radical scavengers from PP is favored at the most intense MW-heating conditions. It has been reported that heating of persimmon slurry under drastic conditions (100–130 °C) greatly enhanced the DPPH^●^ scavenging activity of the corresponding juice; the effect was attributed to a concomitant increase in the content of mono- and oligomeric flavan-3-ols (soluble tannins) as well as of simple phenolic acids like gallic acid and flavonoids like quercetin [46].

In our study, changes in the PJ polar phenol profile and content due to different MW-heating treatments of the pulp were monitored with the aid of UHPLC-DAD-FLD chromatograms at various wavelengths shown in the Supplementary Material (Figure S1). The broad peak corresponding to gallic acid monomer (t_R_ = 1.03 min, identical to that of the standard) dominated the chromatograms at 280 nm. Its content was found to increase greatly from 460 µg/100 mL juice for PJ-C up to 2712 µg/100 mL at the most intense MW-heating conditions (PJ-M120), following the same trend as evidenced for the %RSA values. Other researchers have also reported that gallic acid and its isomeric forms are among the key constituents in the phenol fraction of persimmon juices from various astringent and non-astringent cultivars other than “Jiro” [8,38,46]. Other flavonoid-related compounds with characteristic λmax values at around 350 nm and t_R_ = 8.5–10.5 min became evident in the course of the experiments, showing that PJs of MW-heated PP were also enriched in those constituents. The characteristic fluorescent peaks of catechin and other flavan-3-ols with λexc = 280 nm and λem = 339 nm were also dominant in PJ extracts. Above all, the fluorescent peak at t_R_ = 14.8 min, which was marginally detected in PJ-C, became quite evident in PJ-M60 and PJ-M120. The UV spectral characteristics of this peak (λmax at 274 nm) indicate that it is probably a product of tannin de-polymerization that is formed only after MW-heating of the persimmon pulp for 60 and 120 s (MW energy > 4.2 KJ/g). Altogether, our findings suggest that, under gradually intensified conditions of MW-heating of the persimmon pulp, the polyphenol fraction of PJs is enriched with quercetin or other soluble tannins that are highly active toward the DPPH radicals. A similar effect has been proposed for other pulpy fruits like strawberry and apple [15,16,44].

In the next part of the study, the orange-red solid residue of the MW-heated PP after juice extraction (Figure 1) was considered worthy of further investigation. Our interest was strengthened by two recent studies highlighting that (a) carotenoids are present in high levels in persimmon juice processing residues [9] and (b) persimmon peel contains a lot of low-methoxy acetylated pectin, exhibiting significantly better emulsification properties than commercial citrus pectin [14].

### 3.2. Persimmon Juice Residue

#### 3.2.1. Pectin Content and Structural Features

The pectin content of the solid residues resulting from different juice making treatments is shown in Table 2. At a first look, it can be suggested that PJR-C retained the pectins of untreated PP as the corresponding content values did not differ significantly (11.0 ± 0.7 vs. 12.0 ± 0.9 g/100 g DW respectively). When MW-heating was incorporated into the process as a PP pretreatment step, it resulted in a lower pectin content of the corresponding PJRs, compared with PJR-C (*p* < 0.05). This effect was more pronounced under drastic conditions; PJR-M120 was found to be poorer in total pectin content by almost 18% (9.0 ± 0.9 g/100 g FW). During the proposed hybrid treatment (MW-heating and enzymatic treatment with pectinases), acid-catalysed hydrolysis and depolymerization of solubilized pectin could be induced [48].

**Figure 5:**
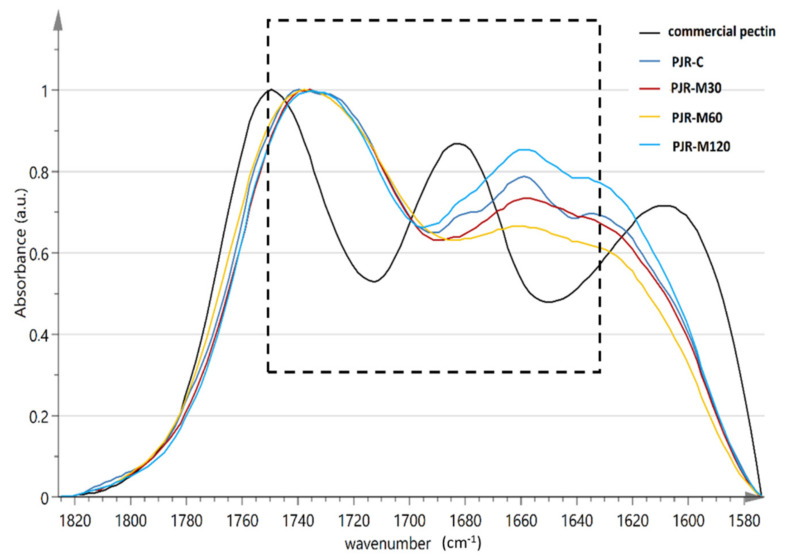
Smoothed, baseline-corrected FT-MIR spectra of pectin isolated from persimmon juice residues obtained from juice production process without (PJR-C) and with pulp treatment at different microwave heating conditions (PJR-M30, PJR-M60, and PJR-M120). The spectrum of commercial low-methoxy pectin from citrus is also shown.

Compared with the two main sources of commercial pectin, i.e., citrus peel and apple pomace, containing 25–35% and 10–15% DW [49], respectively, PJRs of the current study with pectin content ranging from 9.0 to 11.0% demonstrate potential to be valorised as a commercial pectin source. A key factor that determines the functional properties of pectin molecules is the MED, which is the percentage of carboxylic acid moieties that are esterified with methanol. The calculation of this index is not straightforward and usually requires laborious protocols for sample pretreatment to avoid interference from co-existing constituents (water, neutral sugars, proteins, and reagents). The application of non-destructive techniques such as the FT-IR spectroscopy has also been proposed as an alternative, simple means for the routine analysis of pectin from fruits and vegetables. The spectral data may provide accurate measurement of pectin MED even in protein-rich materials, provided that signal pre-processing through deconvolution is applied [50]. In our study, we monitored the structural differences of pectin from different PJRs through smoothed and baseline corrected FT-MIR spectra within the selective region of carboxylic moieties that extends from about 1600 to 1750 cm^−1^, as shown in Figure 5. The absorbance intensity values were first normalized to the maximum intensity of the broad band among 1730–1740 cm^−1^, which is attributed to the bound forms of the carboxylic acid moiety (-COOR). Then, it was more clearly evidenced that pectin extracts from PJRs corresponding to different MW-heating treatments of the PP are differentiated in comparison with PJR-C. Several peaks that are overlapped under the band from 1610 to 1690 cm^−1^ accounted for this finding. More importantly, a shoulder of varying intensity at around 1630 cm^−1^ denoted that differences in the relative concentration of free carboxylate ion groups could contribute to the observed variance among the samples. However, the spectra were poorly resolved. The peak at 1655–1660 cm^−1^ that is due to amide I groups was consistently present in the spectra. Another peak at around 1680 cm^−1^ that is also associated with carbonyl group stretching in unsubstituted amides became evident in the spectra of PJR-C and PJR-M120. By comparison with the respective spectrum of low-methoxy commercial pectin from citrus, it became clear that the target bands at 1730 and at 1600–1630 cm^−1^ spectra could be hardly exploited without further signal processing. Second-order derivatization of the smoothed spectral data helped to overcome the noise and offset effects, so that two negative peaks (valleys) at 1603–1609 and 1622–1627 cm^−1^, together with another two at 1723 and 1733 cm^−1^, were revealed in those spectra. None of them matched with the respective spectrum profile of commercial pectin from citrus, which presented two well-defined peaks at 1603 and 1748 cm^−1^, reflecting possible structural differences of the two different types of pectin [14]. Commercial citrus pectin presented an MED value of 47%, according to the equation A_1750_/(A_1750_ + A_1600_) [32,50]. In the case of PJRs, the exact wavenumbers that were specified in the derivative spectra (around 1730 and 1625 cm^−1^) were used to make a rough estimation of absorbance values that are often claimed to correlate linearly with the MED values from reference measurements. Our results showed that pectins from all the studied PJRs present a relatively low degree of methylesterification, with MED values ranging from 49.4 (PJR-M120) to 51.3% (PJR-M60).

Low methoxy pectins (and their amidated derivatives) are used as food additives (E440) in diet, reduced-sugar products like jams and confectionery products owing to their gelling and thickening properties [48].

#### 3.2.2. Total and Individual Carotenoid Content 

A solid–liquid extraction protocol that has been applied to persimmon peel and pulp in an earlier report [33] was followed to recover carotenoids from the PJRs (Figure 1). Generally, this extraction technique is preferred for laboratory-scale experiments, although its repeatability may be compromised by several factors, e.g., different polarity of the native carotenoids present in the plant tissues or side-reactions during multiple/lengthy extraction steps. Variation in the physical properties of the matrix (e.g., granular size) may also induce large variation in the analytical results. In our study, the dry extract yields from samples of homogenized PP or PJRs varied from approximately 11–12% to almost 30% of the starting material (*w*/*w*, DW). The repeatability of the extraction method (% CV) ranged between ±16.8% (same batch, *n* = 5) and ±24.7% (two different batches, *n* = 4) on a dry weight basis, signifying that this type of error source had to be taken into account in the subsequent assessment study. This limitation is reflected also in the results of Gea-Botella et al. [9], who reported a high standard deviation in individual carotenoid content values of different industrial persimmon juice by-products. Irrespective of this variation, we found that the total carotenoid content of the PJR after heating the PP at the longest MW exposure time/energy conditions (PJR-M120) was higher than that from non-MW-heated pulp (PJR-C) by almost 40% DW. In particular, the corresponding extract contained 246 versus 174 mg/100 g (TCC, as mg β-Cry/100 g, DW). The values are of the same size as those reported for ethanol-acetone-hexane extracts of “Triumph” persimmon pulp residues from industrial juice-processing with no enzymatic aids [9].

Apart from monitoring changes in the total carotenoid content, we put special effort into examining whether and how the different MW-heating conditions of the PP may affect the relative content and native form of individual carotenoids present in the PJR extracts. To the best of our knowledge, only few studies have been carried out on the native carotenoid composition of persimmon fruit tissues [33,34,35,51]. According to the data available, these compounds belong to three broad structural classes; that is, free xanthophylls (FX) consisting of 5,6-epoxy- and hydroxyl- derivatives, hydrocarbon carotenoids (HCs), and xanthophyll fatty acid esters (XE). Among them, β-carotene, lycopene, as well as mono- and di-esters of β-cryptoxanthin, zeaxanthin, and lutein with lauric, myristic, and palmitic acids, along with small amounts of xanthophyll esters with palmitoleic and oleanolic acid, have been structurally elucidated so far [35,51]. The prevalence of β-carotene, β-cryptoxanthin, and its fatty acid mono or diesters is of special importance owing to their pro-vitamin A activity. Moreover, lutein and zeaxanthin along with their esters as well as lycopene are highly appreciated as antioxidants [52]. The structural complexity of those metabolites that is most probably regulated by genetic or environmental factors may differentiate the bioactive potential of the persimmon fruits, as is the case for several natural products [36]. Furthermore, any change in structure during post-harvest processing of the fruits would most probably lead to different biochemical activities or in vivo functions [34].

**Figure 6:**
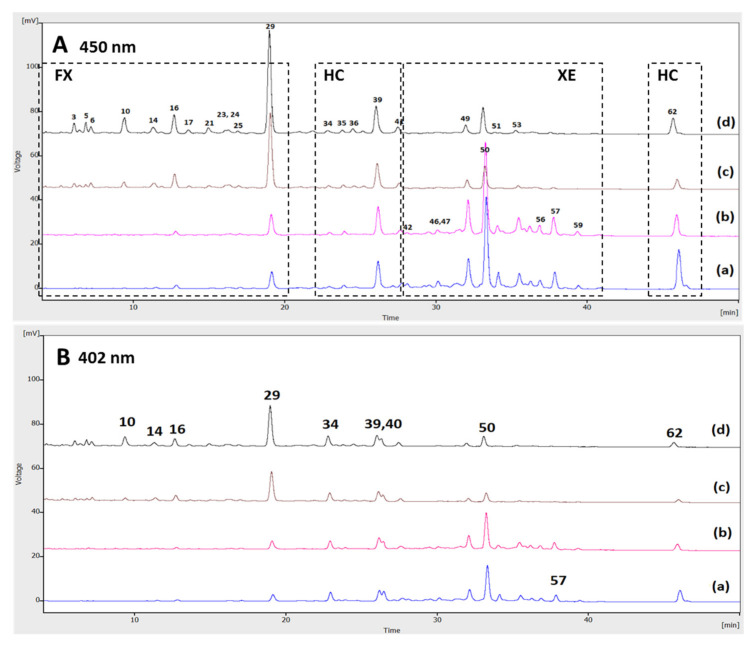
RP-(C30)-HPLC carotenoid profile at (**A**) 450 and (**B**) 402 nm. Chromatographic traces from bottom to the top represent untreated persimmon pulp (PP) (**a**) and persimmon juice residues (PJR) after microwave heating of the pulp for (**b**) 30 s: PJR-M30; (**c**) 60 s: PJR-M60; and (**d**) 120 s: PJR-M120. Each trace was divided in 62 sub-regions based on the elution time intervals of every carotenoid peak detected in the studied extracts. Further information about the elution order and spectral characteristics of the peaks is provided in Appendix A (Table A1).

For testing comparability, we adapted the RP-HPLC separation protocol that has been applied by Cano and co-workers [34,35] in their elaborate study on the characterization of the persimmon carotenoids in free and esterified form through RP-HPLC-PDA-MS/MS. The elution conditions involved separation on a carotenoid C30 column without prior saponification. At a first look, the obtained chromatographic profiles of the untreated PP at 450 nm and 402 nm (Figure 6A(a),B(a)) were identical to those produced by Cano et al. for cv. *Rojo Brillante* fruit tissues [35]. This finding is of special importance considering that the fruits examined here were of the “Jiro” commercial cultivar, the predominant one in the Central Macedonia Region (Greece). Thus, we could assume that the major carotenoids present in the peel and flesh of persimmon fruits from the “Jiro” cultivar are those already reported in the literature to be characteristic of another persimmon cultivar [35]. After MW-heating of the PP and extraction of the juice, the RP-(C30)-HPLC-DAD analysis of the corresponding PJR extracts resulted in the chromatograms shown in Figure 6A(b)–(d),B(b)–(d).

**Figure 7:**
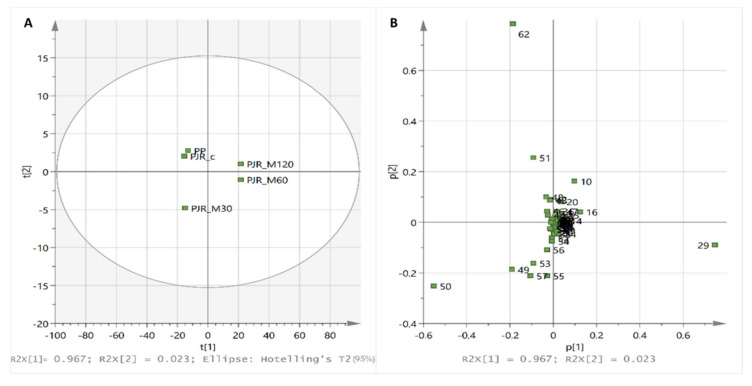
PCA scatterplots of RP-(C30)-HPLC-DAD data at 450 nm representing (**A**) the distribution of persimmon juice residue (PJR) samples according to t1/t2 score values and (**B**) the contribution of individual carotenoids to the scattering of PJR samples. Persimmon pulp (PP) was included as a reference for monitoring the changes in the native carotenoid forms of persimmon pulp.

An overall inspection of the traces indicated that all of the major and minor carotenoid of the PP or PJR extracts were eluted over a time period of 40 min (between 5 and 45 min of each run), within 62 distinct and reproducible time intervals (Δt_R_) that were consecutively numbered, as shown in Appendix A (Table A1); t_R_ values of the eluted compounds within those intervals were found to be shifted by ± 0.1 min to ± 0.6 min from run to run. Greater shifts were evidenced for the less polar constituents that were strongly retained on the column (t_R_ > 33 min). To overcome this type of variation and enhance the interpretability of data, we considered that peak areas within the specific time intervals represent the fingerprint of PP and PJR carotenoids that encompasses variation in the amount and structural characteristics of those compounds as an effect of the MW-heating process under study. The data were then analysed using a multivariate statistical analysis method, that is, principal component analysis (PCA). The normalized peak area values within the specified sub-regions of the chromatograms (no 1 to 62, see Table S2) were mean-centered prior to analysis. Two principal components (PCs) were extracted; the second one explained only 2% of variance in the original data and was considered insignificant for exploratory purposes. Figure 7A,B depict the score distribution of the PJR carotenoid extracts according to the juice processing conditions and in relation to the untreated PP.

The results showed that PJR-M60 and PJR-M120 are clearly differentiated on the basis of their carotenoid profiles and grouped separately from PJRs that resulted from mild or no MW-heating of the pulp. Above all, variance within Δt_R_ **29** and **50** accounted the most for this differentiation. On the other hand, variance within Δt_R_ **49, 51, 53, 55, 57**, and mainly **62** was found to be more informative for the differentiation of PJR-M30 with regard to PJR-C. The latter extract was grouped together with untreated PP, implying that native carotenoids of the pulp remained structurally unaffected during juice processing with no MW-heating.

To obtain clearer information about the identity of those carotenoids, we evaluated the chromatograms in comparison with those of corresponding saponified extracts and performed LC-TOF MS/MS (APCI^+^) analyses that aided in the identification of free xanthophylls (FXs) and hydrocarbons (HCs), as well as certain fatty acid esters (XE), and helped to define the elution time domains, as highlighted in Figure 6A. Additional information can be found in the Supplementary Material (Figure S2). At this stage, existing literature data about the elution order of PP carotenoids on the reverse-phase C30 column were carefully scrutinized [9,34,51,53,54] to extract valuable information. Spectral characteristics and t_R_ values were also compared with those of available standard compounds. As a result, it was confirmed that the two major peaks in Δt_R_ **29** and **50** correspond to (all-*E*)-β-cryptoxanthin (t_R_ = 19 min, λ_max_ = 428, 451, 478 nm) and its mono-laurate ester (t_R_ = 33 min, λ_max_ = 426, 450, 477 nm, *m*/*z* = 735.2 (M + H)^+^, 535 (M + H-12:0)^+^), respectively, while the peak at Δt_R_ **56** represents another β-cryptoxanthin ester, possibly with myristic acid (t_R_ = 36 min, λ_max_ = 451, 478 nm, *m*/*z* = 763.6 [M + H]^+^ and 535.4 [M + H-14:0]^+^). The visible spectral characteristics of the less prominent peaks within intervals no **57** (t_R_ = 37.5, λ_max_ = 425, 451, 478 nm) and **59** (t_R_ = 39, λ_max_ = 451, 475 nm) indicated that they also bear a β-cryptoxanthin moiety. Based on elution order and visible spectral characteristics, the peaks at Δt_R_ **49** (t_R_ = 32 min, λ_max_ = 420, 447, 469 nm), **51** (t_R_ = 33.5 min, λ_max_ = 424, 446, 473 nm), and **53** (t_R_ = 35.3 min, λ_max_ = 424, 449, 473 nm) could be suggested to correspond to fatty acid esters of other existing xanthophylls in PP extracts, like (all-*E*)-lutein and (all-*E*)-zeaxanthin. The free xanthophyll that eluted in Δt_R_ **10** (t_R_ = 9.3 min, λ_max_ = 415, 444, 472 nm, m/z: 585.2 [M + H]^+^) and became more prominent after MW-heating was most probably (all-*E*)-antheraxanthin. The hydrocarbon eluted in Δt_R_ **62** was identified as (all-*E*)-lycopene (t_R_ = 45.7 min, λ_max_ = 446, 472, 503 nm). Noticeably, the peak eluted within Δt_R_ **39** that was identified as (all-*E*)-β-carotene, as well as its isomers that are eluted within Δt_R_ **32** to **41**, were not found to contribute to the observations about the MW-heating effect.

Our findings show that heating of the persimmon pulp at a gradually longer time and more drastic MW energy conditions favored the release of free xanthophylls from their esterified forms. This was also indicated by calculating the % relevant content in Table 3. For example, while xanthophyll esters in PJR-C prevail over their free forms by almost six orders of magnitude, and the same also happens in the case of PJR-M30, this relevant ratio sharply decreased by extending the heating time to 60 s, as the delivered MW energy rose from 0.7 to 4.2 KJ/g. Consequently, the chromatographic profile in the FX region of PJR-M60 became more complex than the one for native carotenoids of PP and a number of small peaks within Δt_R_ **3–6, 17,** and **24** that most probably correspond to epoxycarotenoids were evidenced. Liberation of acids from the plant matrix is known to induce isomerization and rearrangement of 5,6-epoxy- to 5,8-epoxycarotenoids [55], with a characteristic hypsochromic shift in their UV/Vis maxima, as in the case of the peak in Δt_R_ **4** (Table A1). Further intensification of the MW-heating conditions brought about quantitative differences, discussed in the next paragraph. On the other hand, lycopene transformations were induced at the mildest MW-heating conditions of this study (0.7 KJ/g), accounting for pattern differentiation among PJR-M30 and PJR-C. Further investigation of particular carotenoid transformations as a result of various MW-heating conditions of the PP is worthy, but beyond the scope of this article.

Despite the structural changes induced under the most drastic conditions of MW-heating, native fatty acid esters of the PP carotenoids were still present in the PJR-M120. For example, β-Cry laurate was estimated to reach 3098.6 ± 23.3 μg/100 g FW. With regard to the composition in provitamin-A active carotenoids, PJR-M120 was found to be slightly enriched in (all-*E*)-β-carotene, by almost 13% compared with PJR-C (997.4 ± 7.5 μg vs. 880.4 ± 3.5 μg/100 g FW). Summing up the β-cryptoxanthin content in free and major esterified forms resulted in 4687.7 μg/100 g FW, which was almost 16% higher than the respective content of PJR-C (4058.0 ± 17.6 μg/100 g FW). That being the case, the juice residue material after MW-heating of the pulp retained 278.4 ± 2.1 μg retinol activity equivalents (RAE) [56], higher than the respective value found in PJR-C (242.4 ±1.0 μg RAE/100 g FW). These values were of the same size as those reported by Cano and co-workers for persimmon peel tissues [35].

## 4. Conclusions

For the first time, integration of MW-heating into the pretreatment step of persimmon juice processing was studied. Taking together the results on multiple quality characteristics, we can conclude that MW-heating of the persimmon pulp at the mildest energy conditions employed (0.7 kJ/g) resulted in juice and residue that were both of inferior quality. Incomplete inactivation of enzymes combined with partial cellular disruption and liberation of acids could be a possible reason for this outcome. A higher yield of juice with improved microbiological quality and nutritional value was achieved when PP was MW-heated at the most drastic conditions (8.4 kJ/g). The juice was enriched in gallic acid, polyphenols, and potent DPPH^●^ scavengers, but its orange color faded and was more acidic, which may compromise its acceptability by an average consumer. In the current study, sensory analysis was not carried out owing to COVID-19 restrictions. In any case, prior to upscaling of the proposed process, the shelf life of the PJ and its sensorial appreciation need to be investigated. Our findings support the exploitation of the juice residue of the MW-heated PP as an excellent source of provitamin-A active carotenoids and of low-methoxy pectin. Moreover, the recovered fractions of carotenoids from PJRs could be used as natural colorants of the juice. This potential could counterbalance the additional cost of the microwave unit operation and enhance the profitability of the process.

## Figures and Tables

**Table 1:** Yield and physicochemical parameters of persimmon juice produced without (PJ-C) and after persimmon pulp treatment at different microwave heating conditions (PJ-M30, PJ-M60, PJ-M120).

	PJ-C	PJ-M30	PJ-M60	PJ-M120
Yield (% *w*/*w*)	36.6 ± 1.5 ^a^	72.1 ± 4.8 ^b,c^	67.0 ± 3.7 ^b,c^	71.8 ± 1.5 ^b,c^
Total soluble solids (°Brix)	15.50 ± 0.32 ^b^	13.42 ± 0.59 ^a^	15.17 ± 0.82 ^b^	15.17 ± 0.26 ^b^
Sugars				
Glucose (g/L)	74.43 ± 7.28 ^a^	65.65 ± 6.40 ^a^	66.25 ± 6.32 ^a^	80.30 ± 1.10 ^a^
Fructose (g/L)	78.24 ± 8.37 ^a^	80.52 ± 4.49 ^a^	82.58 ± 4.49 ^a^	91.12 ± 1.16 ^a^
Glucose-to-Fructose Ratio	0.95 ± 0.01 ^c^	0.81 ± 0.03 ^a^	0.80 ± 0.03 ^a^	0.88 ± 0.01 ^b^
pH	3.12 ± 0.01 ^a^	2.91 ± 1.23 ^a^	3.32 ± 0.06 ^a^	3.40 ± 0.03 ^a^
Total Acidity (g malic acid/100 mL)	0.57 ± 0.03 ^a^	0.69 ± 0.04 ^b^	0.67 ± 0.04 ^b^	0.71 ± 0.03 ^b^
Organic Acids				
Acetic acid (g/L)	6.04 ± 0.15 ^a^	6.60 ± 0.12 ^b^	8.33 ± 0.25 ^c^	8.62 ± 0.27 ^c^
Citric acid (g/L)	1.19 ± 0.01 ^a^	1.80 ± 0.02 ^d^	1.59 ± 0.02 ^c^	1.49 ± 0.02 ^b^
Tartaric acid (g/L)	2.25 ± 0.03 ^b^	3.59 ± 0.05 ^c^	2.29 ± 0.04 ^b^	0.50 ± 0.01 ^a^
Malic acid (g/L)	1.84 ± 0.02 ^b^	2.86 ±0.03 ^c^	1.88 ± 0.02 ^b^	0.49 ± 0.01 ^a^
L	55.61 ± 0.01 ^a^	57.90 ± 0.31 ^b^	58.78 ± 0.71 ^c^	61.70 ± 0.14 ^d^
a*	2.59 ± 0.06 ^c^	−0.16 ± 0.08 ^b^	−0.24 ± 0.06 ^b^	−1.69 ± 0.05 ^a^
b*	20.44 ± 8.61 ^b^	18.47 ± 1.27 ^b^	18.01 ± 0.53 ^b^	12.19 ± 0.14 ^a^
C*	20.72 ± 8.24 ^b,c^	18.47 ± 1.27 ^b^	18.01 ± 0.53 ^b^	12.30 ± 0.14 ^a^
h	77.80 ± 14.80 ^a^	90.49 ± 0.21 ^b^	90.77 ± 0.21 ^b^	97.88 ± 0.22 ^c^
ΔE*	0	4.08 ± 0.57 ^a^	4.90 ± 0.26 ^a^	11.11 ± 0.41 ^b^

All values are shown as the mean ± standard deviation (*n* = 3). In each row, different superscript letters indicate significant differences (*p* ≤ 0.05). $C^{*}=\sqrt{a^{*}{}^{2}+b^{*}{}^{2}}$, ho*=tan−1a*b* and $\Delta E_{\mathrm{ab}}^{*}=\sqrt{{\left(\Delta L\right)}^{2}+{\left(\Delta a^{*}\right)}^{2}+{\left(\Delta b^{*}\right)}^{2}}$ values expressed in angle degrees.

**Table 2:** Pectin content of untreated persimmon pulp (PP) and persimmon juice residues (PJR) obtained from juice production process without (PJR-C) or with pulp treatment at different microwave heating conditions (PJR-M30, PJR-M60, and PJR-M120).

**Sample**	**Pectin Content (g/100 g DW)**
PP	12.0 ± 0.9 ^c^
PJR-C	11.0 ± 0.7 ^b,c^
PJR-M30	10.0 ± 0.9 ^a,b^
PJR-M60	9.0 ± 0.9 ^a^
PJR-M120	9.0 ± 0.9 ^a^

All values are shown as the mean ± standard deviation (*n* = 3) and are expressed on a dry weight basis. Different superscript letters indicate significant differences (*p* ≤ 0.05).

**Table 3:** Relative content of free xanthophylls (FXs), xanthophyll esters (XEs), and hydrocarbon carotenoids (HCs) as well as unidentified carotenoids (U) in untreated persimmon pulp (PP) and persimmon juice residues (PJR) obtained from juice production process without (PJR-C) or with pulp treatment at different microwave heating conditions (PJR-M30, PJR-M60, and PJR-M120).

	PP	PJR-C	PJR-M30	PJR-M60	PJR-M120
	Carotenoid content [% of total peak area at 450 nm]				
FX	13.4 ± 2.5	9.2 ± 1.4	10.8 ± 1.4	51.9 ± 2.9	55.9 ± 0.5
XE	53.7 ± 2.7	57.6 ± 2.6	61.2 ± 3.2	15.8 ± 2.0	14.2 ± 0.3
HC	27.8 ± 1.4	27.6 ± 2.4	22.4 ± 0.4	22.1 ± 1.1	22.3 ± 0.2
u	5.5 ± 1.3	4.8 ± 1.1	5.2 ± 2.4	7.7 ± 1.4	7.7 ± 0.3

All values are shown as mean ± standard deviation (*n* = 3).

## Supplementary Materials

The following are available online at https://www.mdpi.com/article/10.3390/foods10112650/s1. Table S1. Microwave heating conditions in the preliminary tests. Figure S1. RP UHPLC-DAD-FL Chromatograms of polar phenol extracts of persimmon juices produced without (PJ-C) and after persimmon pulp treatment at different microwave heating conditions (PJ-M30, PJ-M60, PJ-M120) at (a) 280 nm (UV), (b) 280 nm (exc.)/320 nm (em.) (hydroxycinnamic acid derivatives), and (c) 280 nm (exc.)/339 nm (em.) (catechin analogues) (FL). UV/Vis spectra of peaks highlighted at the flavonoids are also given (d). Additional data about the identification of xanthophyll esters in PP and PJR extracts. Text and Figure S2. Changes in the RP-HPLC profile of PP carotenoid extract at 450 nm after saponification.

## Abbreviations

AMCs	Aerobic mesophilic counts
DPPH^●^	1,1-diphenyl-2-picrylhydrazyl radical
F-C	Folin–Ciocalteu
GAE	Gallic acid equivalents
MW	Microwave
PJ	Persimmon juice
PJR	Persimmon juice residue
PP	Persimmon pulp
RAE	Retinol activity equivalents
TA	Titratable acidity
YMCs	Yeasts and molds counts
β-Car	β-carotene
β-Cry	β-cryptoxanthin

## Appendix A

**Table A1:** Carotenoid elution order on reverse phase-C30 column, visible, and mass spectral characteristics of peaks (S/N > 3) that presented the carotenoid-specific fine structure in the spectral region between λ=410−470 nm and possible identity based also on the comparison with standard compounds and literature data [9,34,51,53,54].

*No*	*Peak Elution Time* *Interval,* Δ*t_R_ (min) ^1^*	*λ_max_ (nm)*	*Possible Identity*
*1*	5.14–5.24	407	FX, ni ^2^
*2*	5.48–5.55	426	FX, ni
*3*	5.99–6.16	(328),415,440,470	FX, ni
*4*	6.30–6.45	(389), 412,426,450	(all-E)-Luteoxanthin
*5*	6.78–6.92	415,439,469	(all-E)-Violaxanthin
*6*	7.09–7.28	414,432,464	FX, ni
*7*	7.85–8.08	(340),414,439,469	FX, ni
*8*	8.11–8.35	404,422,450	FX, ni
*9*	8.53–8.68	410,436,467	FX, ni
*10*	9.20–9.45	415,444,472	(all-E)-Antheraxanthin
*11*	9.73–9.93	398,419,442	FX, ni
*12*	10.05–10.18	397,420,442	FX, ni
*13*	10.57–10.72	432,451	FX, ni
*14*	11.18–11.45	422,448,473	(all-E)-Lutein
*15*	11.68–11.91	426	FX, ni
*16*	12.49–12.83	423,451,477	(all-E)-Zeaxanthin
*17*	13.30–13.69	420, 442, 469	5,6-epoxy- Lutein
*18*	14.01–14.34	429,453	FX, ni
*19*	14.34–14.61	443,473	FX, ni
*20*	14.85–15.09	418,445,473	FX, ni
*21*	15.16–15.28	410,443,470	FX, ni
*22*	15.41–16.00	335,423,450,467	FX, ni
*23*	15.93–16.13	335,423,449,467	FX, ni
*24*	15.98–16.33	334,442,469	Unknown+5,6-epoxy- β-Cryptoxanthin
*25*	16.74–17.05	450	FX, ni
*26*	17.16–17.93	440	FX, ni
*27*	17.50–17.88	440	FX, ni
*28*	18.34–18.64	421,446	FX, ni
*29*	18.81–19.06	(428),451,478	(all-E)-β-Cryptoxanthin
*30*	19.85–20.39	440	FX, ni
*31*	20.55–21.05	450,475	ni
*32*	21.51–22.03	(327),446,473	ni
*33*	22.19–22.60	450	ni
*34*	22.72–22.95	(359),379,399,423	Luteoxanthin or Neoxanthin ester
*35*	23.70–23.93	335,421,449,473	HC, ni
*36*	24.28–24.71	(330),421,446,472	ni
*37*	24.85–25.23	454,470	ni
*38*	25.01–25.47	450	ni
*39*	25.89–26.19	426,452,478	(all-E)-β-carotene
*40*	26.51–27.19	419,438,469	violaxanthin ester
*41*	26.98–27.81	(322),420,445,470	(9Z)-β-carotene
*42*	27.61–28.11	418,441,469	XE, ni
*43*	27.89–28.87	425,441,473	XE, ni
*44, 45*	28.96–29.82	329,434,465	XE, ni
*46, 47*	29.75–30.74	(394),418,441,468	XE, ni
*48*	31.08–32.06	(329),412,438,465	XE, ni
*49*	31.82–32.21	420,447,469	(all-E)-zeaxanthin palmitate
*50*	32.33–33.40	426,450,477	(all-E)-β-cryptoxanthin laurate
*51*	32.94–34.20	424,446,473	(all-E)-lutein 3-O-palmitate
*52*	33.70–34.60	(311),418,445,469	XE, ni
*53*	35.09–35.59	424,449,473	(all-E)-zeaxanthin myristate
*54*	35.36–35.99	(334),450,477	XE, ni
*55*	35.85–36.33	420,446,473	(all-E)-antheraxanthin ester
*56*	36.07–36.96	451,478	(all-E)-β-cryptoxanthin myristate
*57*	37.34–38.38	(425),451,478	XE, ni
*58*	38.12–38.63	452,474	XE, ni
*59*	38.94–39.49	451,475	XE, ni
*60*	39.53–40.17	450,469	XE, ni
*61*	40.14–40.98	450,469	XE, ni
*62*	45.33–46.12	446,472,503	lycopene

**^1^** The intervals between maximum and minimum elution time of each carotenoid peak among replicate runs were calculated after alignment of chromatographic traces relative to the major peak of PP extract that eluted at t_R_ ~ 33.2 min and corresponds to the lauric acid monoester of (all‒*E*)‒β‒cryptoxanthin. **^2^** ni: non identified

## References

[1] Llácer G., Badenes M.L. (2002). Persimmon production and market. Options Mediterr..

[2] Butt M.S., Sultan M.T., Aziz M., Naz A., Ahmed W., Kumar N., Imran M. (2015). Persimmon (*Diospyros kaki*) fruit: Hidden phytochemicals and health claims. EXCLI J..

[3] Food and Agriculture Organization Corporate Statistical Database (FAOSTAT) Crops. http://www.fao.org/faostat/en/#data/QC.

[4] Pastopoulos S., Kazantzis K., Marsanidis S. (2017). The cultivation of Persimmon in Yianitsa region (in Greek). Agrotypos.

[5] Ordoudi S.A., Bakirtzi C., Tsimidou M.Z. (2018). The potential of tree fruit stone and seed wastes in Greece as sources of bioactive ingredients. Recycling.

[6] Curi P.N., Tavares B.S., Almeida A.B., Pio R., Pasqual M., Peche P.M., de Souza V.R. (2017). Characterization and influence of subtropical persimmon cultivars on juice and jelly characteristics. An. Acad. Bras. Cienc..

[7] González E., Vegara S., Martí N., Valero M., Saura D. (2015). Physicochemical characterization of pure persimmon juice: Nutritional quality and food acceptability. J. Food Sci..

[8] Jiménez-Sánchez C., Lozano-Sánchez J., Marti N., Saura D., Valero M., Segura-Carretero A., Fernández-Gutiérrez A. (2015). Characterization of polyphenols, sugars, and other polar compounds in persimmon juices produced under different technologies and their assessment in terms of compositional variations. Food Chem..

[9] Gea-Botella S., Agulló L., Martí N., Martínez-Madrid M.C., Lizama V., Martín-Bermudo F., Berná G., Saura D., Valero M. (2021). Carotenoids from persimmon juice processing. Food Res. Int..

[10] Jiménez-Sánchez C., Lozano-Sánchez J., Segura-Carretero A., Fernández-Gutiérrez A. (2017). Alternatives to conventional thermal treatments in fruit-juice processing. Part 2: Effect on composition, phytochemical content, and physicochemical, rheological, and organoleptic properties of fruit juices. Crit. Rev. Food Sci. Nutr..

[11] Meléndez-Martínez A.J., Mandić A.I., Bantis F., Böhm V., Borge G.I.A., Brnčić M., Bysted A., Cano M.P., Dias M.G., Elgersma A. (2020). A comprehensive review on carotenoids in foods and feeds: Status quo, applications, patents, and research needs. Crit. Rev. Food Sci. Nutr..

[12] Markets and Markets (2019). Carotenoids Market by Type, Application, Region—Global Forecast 2026|COVID-19 Impact on Carotenoids Market.

[13] UN General Assembly (2015). Transforming Our World: The 2030 Agenda for Sustainable Development.

[14] Jiang Y., Xu Y., Li F., Li D., Huang Q. (2020). Pectin extracted from persimmon peel: A physicochemical characterization and emulsifying properties evaluation. Food Hydrocoll..

[15] Guo Q., Sun D.W., Cheng J.H., Han Z. (2017). Microwave processing techniques and their recent applications in the food industry. Trends Food Sci. Technol..

[16] Jiménez-Sánchez C., Lozano-Sánchez J., Segura-Carretero A., Fernández-Gutiérrez A. (2017). Alternatives to conventional thermal treatments in fruit-juice processing. Part 1: Techniques and applications. Crit. Rev. Food Sci. Nutr..

[17] Gerard K.A., Roberts J.S. (2004). Microwave heating of apple mash to improve juice yield and quality. LWT-Food Sci. Technol..

[18] Cendres A., Chemat F., Page D., Le Bourvellec C., Markowski J., Zbrzezniak M., Renard C.M.G.C., Plocharski W. (2012). Comparison between microwave hydrodiffusion and pressing for plum juice extraction. LWT-Food Sci. Technol..

[19] Pérez-Grijalva B., Herrera-Sotero M., Mora-Escobedo R., Zebadúa-García J.C., Silva-Hernández E., Oliart-Ros R., Pérez-Cruz C., Guzmán-Gerónimo R. (2018). Effect of microwaves and ultrasound on bioactive compounds and microbiological quality of blackberry juice. LWT-Food Sci. Technol..

[20] Chutia H., Mahanta C.L. (2021). Green ultrasound and microwave extraction of carotenoids from passion fruit peel using vegetable oils as a solvent: Optimization, comparison, kinetics, and thermodynamic studies. Innov. Food Sci. Emerg. Technol..

[21] Papapostolou M., Mantzouridou F.T., Tsimidou M.Z. (2021). Flavored olive oil as a preservation means of reduced salt spanish style green table olives (Cv. chalkidiki). Foods.

[22] Lalou S., Hatzidimitriou E., Papadopoulou M., Kontogianni V.G., Tsiafoulis C.G., Gerothanassis I.P., Tsimidou M.Z. (2015). Beyond traditional balsamic vinegar: Compositional and sensorial characteristics of industrial balsamic vinegars and regulatory requirements. J. Food Compos. Anal..

[23] Horwitz W. (2003). Official Methods of Analysis of Association of Official Analytical Chemists International Method No. 982.27.

[24] Ordoudi S.A., Mantzouridou F., Daftsiou E., Malo C., Hatzidimitriou E., Nenadis N., Tsimidou M.Z. (2014). Pomegranate juice functional constituents after alcoholic and acetic acid fermentation. J. Funct. Foods.

[25] Cheng C.X., Jia M., Gui Y., Ma Y. (2020). Comparison of the effects of novel processing technologies and conventional thermal pasteurisation on the nutritional quality and aroma of Mandarin (*Citrus unshiu*) juice. Innov. Food Sci. Emerg. Technol..

[26] AOAC (2006). AOAC official method 967.21 Ascorbic Acid in Vitaimin Prepaprations and juices 2,6-Dichloroindophenol titremetric method. Official Methods of Analysis.

[27] Ubeda C., Callejón R.M., Hidalgo C., Torija M.J., Mas A., Troncoso A.M., Morales M.L. (2011). Determination of major volatile compounds during the production of fruit vinegars by static headspace gas chromatography-mass spectrometry method. Food Res. Int..

[28] Huang D., Boxin O.U., Prior R.L. (2005). The chemistry behind antioxidant capacity assays. J. Agric. Food Chem..

[29] Tsimidou M.Z., Sotiroglou M., Mastralexi A., Nenadis N., García-González D.L., Toschi T.G. (2019). In house validated UHPLC protocol for the determination of the total hydroxytyrosol and tyrosol content in virgin olive oil fit for the purpose of the health claim introduced by the EC Regulation 432/2012 for “olive oil polyphenols”. Molecules.

[30] Chang Y.L., Lin J.T., Lin H.L., Liao P.L., Wu P.J., Yang D.J. (2019). Phenolic compositions and antioxidant properties of leaves of eight persimmon varieties harvested in different periods. Food Chem..

[31] Godoy-Caballero M.P., Acedo-Valenzuela M.I., Galeano-Díaz T. (2012). Simple quantification of phenolic compounds present in the minor fraction of virgin olive oil by LC-DAD-FLD. Talanta.

[32] Manrique G.D., Lajolo F.M. (2002). FT-IR spectroscopy as a tool for measuring degree of methyl esterification in pectins isolated from ripening papaya fruit. Postharvest Biol. Technol..

[33] Bordiga M., Travaglia F., Giuffrida D., Mangraviti D., Rigano F., Mondello L., Arlorio M., Coïsson J.D. (2019). Characterization of peel and pulp proanthocyanidins and carotenoids during ripening in persimmon “Kaki Tipo” cv, cultivated in Italy. Food Res. Int..

[34] Cano M.P., Gómez-Maqueo A., Fernández-López R., Welti-Chanes J., García-Cayuela T. (2019). Impact of high hydrostatic pressure and thermal treatment on the stability and bioaccessibility of carotenoid and carotenoid esters in astringent persimmon (*Diospyros kaki* Thunb, var. Rojo Brillante). Food Res. Int..

[35] Cano M.P., Gómez-Maqueo A., Welti-Chanes J., García-Cayuela T. (2018). Characterization of carotenoid and carotenoid esters of astringent persimmon tissues (*Diospyros kaki* Thunb. var. Rojo brillante). Effects of thermal and high pressure non-thermal processing M. Preprints.

[36] Granado-Lorencio F., Olmedilla-Alonso B., Herrero-Barbudo C., Blanco-Navarro I., Pérez-Sacristán B., Blázquez-García S. (2007). In vitro bioaccessibility of carotenoids and tocopherols from fruits and vegetables. Food Chem..

[37] Veberic R., Jurhar J., Mikulic-Petkovsek M., Stampar F., Schmitzer V. (2010). Comparative study of primary and secondary metabolites in 11 cultivars of persimmon fruit (*Diospyros kaki* L.). Food Chem..

[38] Lee J.H., Lee Y.B., Seo W.D., Kang S.T., Lim J.W., Cho K.M. (2012). Comparative studies of antioxidant activities and nutritional constituents of persimmon juice (*Diospyros kaki* L. cv. Gapjubaekmok). Prev. Nutr. Food Sci..

[39] Koskiniemi C.B., Truong V.D., McFeeters R.F., Simunovic J. (2013). Quality evaluation of packaged acidified vegetables subjected to continuous microwave pasteurization. LWT-Food Sci. Technol..

[40] Cendres A., Chemat F., Maingonnat J.F., Renard C.M.G.C. (2011). An innovative process for extraction of fruit juice using microwave heating. LWT-Food Sci. Technol..

[41] Stinco C.M., Szczepańska J., Marszałek K., Pinto C.A., Inácio R.S., Mapelli-Brahm P., Barba F.J., Lorenzo J.M., Saraiva J.A., Meléndez-Martínez A.J. (2019). Effect of high-pressure processing on carotenoids profile, colour, microbial and enzymatic stability of cloudy carrot juice. Food Chem..

[42] European Commission (2005). COMMISSION REGULATION (EC) No 2073/2005 of 15 November 2005 on microbiological criteria for foodstuffs. Off. J. Eur. Union.

[43] Mendes-Oliveira G., Deering A.J., San Martin-Gonzalez M.F., Campanella O.H. (2020). Microwave pasteurization of apple juice: Modeling the inactivation of Escherichia coli O157:H7 and Salmonella Typhimurium at 80–90 °C. Food Microbiol..

[44] Zhang S., Zhang R. (2014). Effects of Microwave Pretreatment of Apple Raw Material on the Nutrients and Antioxidant Activities of Apple Juice. J. Food Process..

[45] Dars A.G., Hu K., Liu Q., Abbas A., Xie B., Sun Z. (2019). Effect of thermo-sonication and ultra-high pressure on the quality and phenolic profile of mango juice. Foods.

[46] Lee D.W., Lee S.C. (2012). Effect of heat treatment condition on the antioxidant and several physiological activities of non-astringent persimmon fruit juice. Food Sci. Biotechnol..

[47] Siguemoto É.S., Pereira L.J., Gut J.A.W. (2018). Inactivation Kinetics of Pectin Methylesterase, Polyphenol Oxidase, and Peroxidase in Cloudy Apple Juice under Microwave and Conventional Heating to Evaluate Non-Thermal Microwave Effects. Food Bioprocess Technol..

[48] Cui J., Zhao C., Feng L., Han Y., Du H., Xiao H., Zheng J. (2021). Pectins from fruits: Relationships between extraction methods, structural characteristics, and functional properties. Trends Food Sci. Technol..

[49] Sundarraj A.A., Ranganathan T.V. (2017). A Review -Pectin from Agro and Industrial Waste. Int. J. Appl. Environ. Sci. ISSN.

[50] Kyomugasho C., Christiaens S., Shpigelman A., Van Loey A.M., Hendrickx M.E. (2015). FT-IR spectroscopy, a reliable method for routine analysis of the degree of methylesterification of pectin in different fruit- and vegetable-based matrices. Food Chem..

[51] Hitaka Y., Nakano A., Tsukigawa K., Manabe H., Nakamura H., Nakano D., Kinjo J., Nohara T., Maeda H. (2013). Characterization of carotenoid fatty acid esters from the peels of the persimmon *Diospyros kaki*. Chem. Pharm. Bull..

[52] Marangoni A.G., Mercadante A.Z. (2019). Carotenoid Esters in Foods Food Chemistry, Function and Analysis.

[53] Zhou C., Zhao D., Sheng Y., Tao J., Yang Y. (2011). Carotenoids in fruits of different persimmon cultivars. Molecules.

[54] Giordani E., Doumett S., Nin S., Del Bubba M. (2011). Selected primary and secondary metabolites in fresh persimmon (Diospyros kaki Thunb.): A review of analytical methods and current knowledge of fruit composition and health benefits. Food Res. Int..

[55] Saini R.K., Keum Y.S. (2018). Carotenoid extraction methods: A review of recent developments. Food Chem..

[56] Trumbo P., Yates A.A., Schlicker S., Poos M. (2001). Dietary Reference Intakes: Vitamin A, Vitamin K, Arsenic, Boron, Chromium, Copper, Iodine, Iron, Manganese, Molybdenum, Nickel, Silicon, Vanadium, and Zinc. J. Am. Diet. Assoc..

