# Supplementary material for: On the Effect of Microwave Heating on Quality Characteristics and Functional Properties of Persimmon Juice and Its Residue
Source: Foods. 2021 Nov 1;10(11):2650. doi: 10.3390/foods10112650 (PMC8624191; doi:10.3390/foods10112650)
Supplement: Supplementary file 1 [file foods-10-02650-s001.zip › foods-1423901-supplementary.pdf]

On the Effect of Microwave Heating on Quality Characteristics and Functional Properties of Persimmon Juice and Its Residue

Sofia Lalou [1], Stella A. Ordoudi [1,2,*], and Fani Th. Mantzouridou [1,2,*]

[1] Laboratory of Food Chemistry and Technology, School of Chemistry, Aristotle University of Thessaloniki, 54124 Thessaloniki, Greece; lalousofia@gmail.com
[2] Natural Products Research Center of Excellence (NatPro-AUTH), Center for Interdisciplinary Research and Innovation (CIRI-AUTH), 57001 Thessaloniki, Greece
* Correspondence: steord@chem.auth.gr (S.A.O.); fmantz@chem.auth.gr (F.T.M.); Tel.: (+30) 2310-997847 (S.A.O); (+30) 2310-997774 (F.T.M)

Supplementary materials

Table S1. Microwave heating conditions in the preliminary tests.

Power (Watt)	*Heating time (s)*	*Microwave energy* (kJ/g) *	*Final Temperature (°C)*
210	30	2.1	30
	60	4.2	45
	90	6.3	73
	120	8.4	80
350	30	3.5	40
	60	7	62
	90	10.5	90
	120	14	101
700	30	7	55
	60	14	87
	90	21	99
	120	28	105

** Microwave energy was calculated using the equation E= Wt/m, where W is microwave oven power; t is time of microwave exposure, and m equals the quantity of sample (Pérez-Grijalva et al., 2018)*

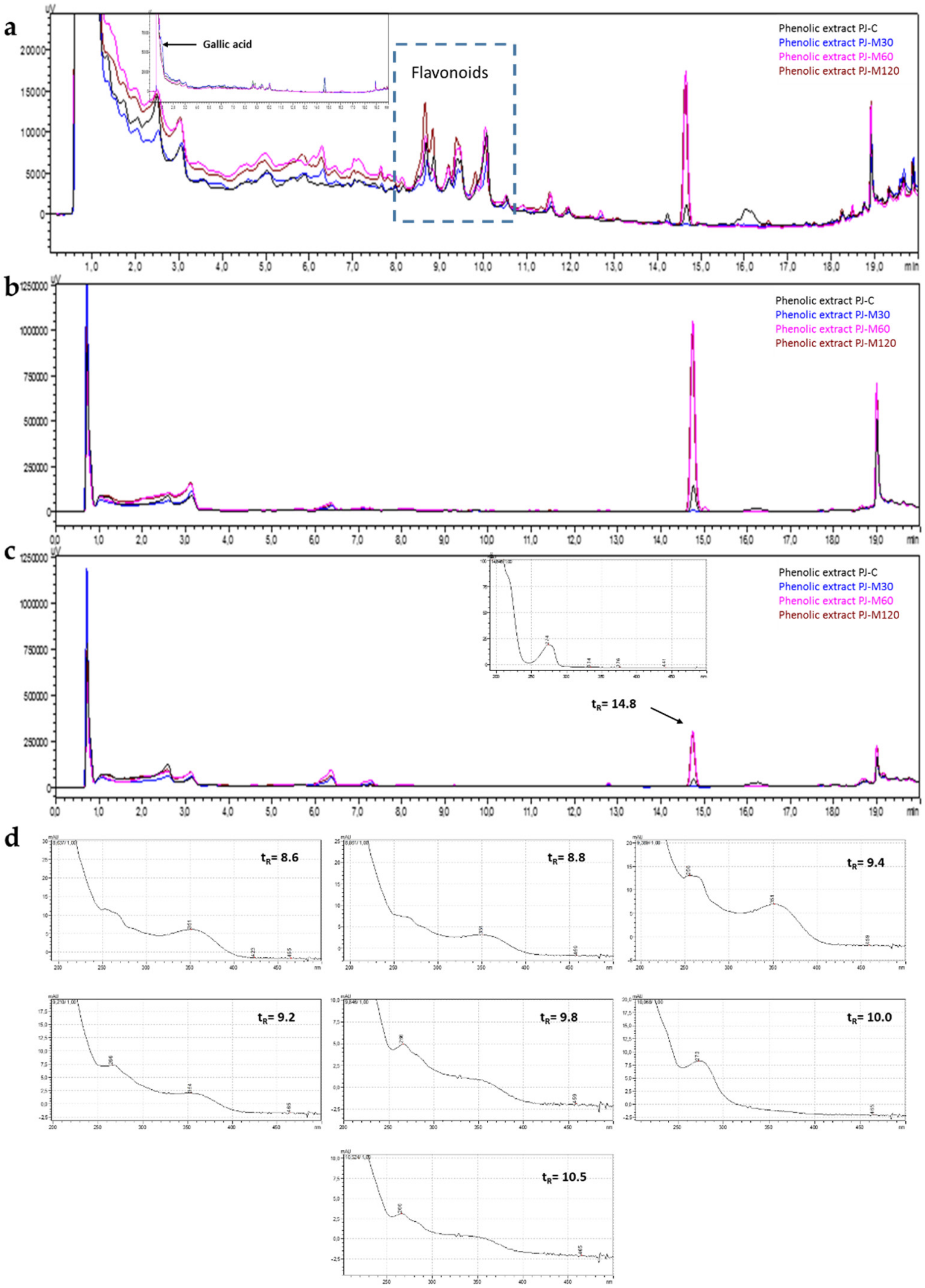

Figure S1. RP UHPLC-DAD-FL Chromatograms of polar phenol extracts of persimmon juices produced without (PJ-C) and after persimmon pulp treatment at different microwave heating conditions (PJ-M30, PJ-M60, PJ-M120) at (a) 280 nm (UV), (b) 280 nm (exc.)/ 320 nm (em.) (hydroxycinnamic acid derivatives) and(c) 280 nm (exc.)/339 nm (em.) (catechin analogues) (FL). UV-Vis spectra of peaks highlighted at the flavonoids are also given (d).

Additional data about the identification of xanthophyll esters in PP and PJR extracts

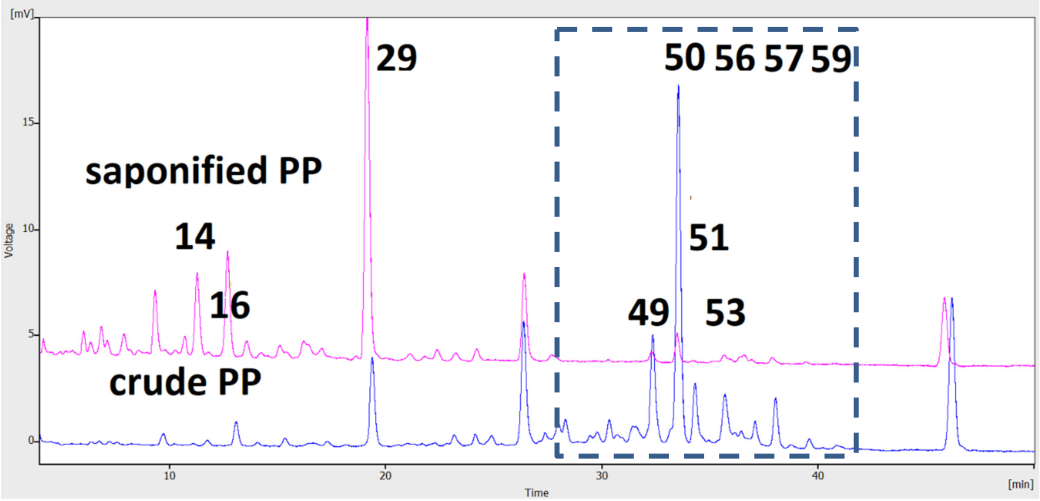

Figure S2. Changes in the RP-HPLC profile of PP carotenoid extract at 450 nm after saponification according to the protocol described below.The region of xanthophyll esters is highlighted.

Saponification of carotenoids was conducted following the protocol of Cano et al. 2019[1].

Briefly, 4 mL of 300 g/L methanolic KOH solution was added to the dried organic phase in nitrogen atmosphere and magnetically stirred for 2h in the dark. Next, the saponified extract was transferred to a funnel containing 15 mL of diethyl ether and was washed five times with 25 mL of water saturated with NaCl. The aqueous phase was discarded each time until neutralization of the pH. The organic layer/ extract. The organic layer was first dried over anhydrous sodium sulphate for 10 min and then evaporated to dryness in a rotary evaporator (35 ^{o}C). Prior to injection, the carotenoid–rich fraction was dissolved in 2 mL of a mixture of MeOH:MTBE:H2O (45.5: 52.5: 2 v/v/v) and filtered through a 0.45 µm pore membrane filter.

Carotenoid analyses by LC-QTOF-MS/MS (APCI^{+})

A liquid chromatography-high resolution tandem mass spectrometry system was used for the analysis of the extracts consisting of an ExionAC HPLC system (solvent degasser, two pumps, autosampler, column oven, controller) and a X500R quadrupole-Time of Flight (QTOF) mass spectrometer (SCIEX, Framingham, MA, USA). Sample acquisition and processing was carried out by SCIEX OS ver 1.7. The mass spectrometer settings were adopted from [1]: Nebulizer gas was set at 30 psi and curtain gas at 25 psi (both nitrogen). Desolvation temperature was set at 400 ^{o}C and nubulizer (corona) current at 4 µA. Declustering potential was set at 60V. MS/MS spectra were collected at 35V with an energy spread of 15V. MS data were collected from 100-1200 Da. The chromatographic conditions were identical to those applied in HPLC-DAD analyses.

.

Supplementary References

1. Cano, M.P.; Gómez-Maqueo, A.; Fernández-López, R.; Welti-Chanes, J.; García-Cayuela, T. Impact of high hydrostatic pressure and thermal treatment on the stability and bioaccessibility of carotenoid and carotenoid esters in astringent persimmon (Diospyros kaki Thunb, var. Rojo Brillante). *Food Res. Int.* **2019**, *123*, 538–549.
